# The modified patient enablement instrument: a Portuguese cross-cultural adaptation, validity and reliability study

**DOI:** 10.1038/npjpcrm.2016.87

**Published:** 2017-01-12

**Authors:** Mafalda Remelhe, Pedro M Teixeira, Irene Lopes, Luís Silva, Jaime Correia de Sousa

**Affiliations:** 1 School of Health Sciences, University of Minho, Braga, Portugal; 2 Life and Health Sciences Research Institute (ICVS), School of Health Sciences, University of Minho, Braga, Portugal; 3 Oceanos Family Health Unit, Porto, Portugal; 4 Horizonte Family Health Unit, Porto, Portugal

## Abstract

Enabling patients with asthma to obtain the knowledge, confidence and skills they need in order to assume a major role in the management of their disease is cost effective. It should be an integral part of any plan for long-term control of asthma. The modified Patient Enablement Instrument (mPEI) is an easily administered questionnaire that was adapted in the United Kingdom to measure patient enablement in asthma, but its applicability in Portugal is not known. Validity and reliability of questionnaires should be tested before use in settings different from those of the original version. The purpose of this study was to test the applicability of the mPEI to Portuguese asthma patients after translation and cross-cultural adaptation, and to verify the structural validity, internal consistency and reproducibility of the instrument. The mPEI was translated to Portuguese and back translated to English. Its content validity was assessed by a debriefing interview with 10 asthma patients. The translated instrument was then administered to a random sample of 142 patients with persistent asthma. Structural validity and internal consistency were assessed. For reproducibility analysis, 86 patients completed the instrument again 7 days later. Item-scale correlations and exploratory factor analysis were used to assess structural validity. Cronbach’s alpha was used to test internal consistency, and the intra-class correlation coefficient was used for the analysis of reproducibility. All items of the Portuguese version of the mPEI were found to be equivalent to the original English version. There were strong item-scale correlations that confirmed construct validity, with a one component structure and good internal consistency (Cronbach’s alpha >0.8) as well as high test–retest reliability (ICC=0.85). The mPEI showed sound psychometric properties for the evaluation of enablement in patients with asthma making it a reliable instrument for use in research and clinical practice in Portugal. Further studies are needed to confirm its responsiveness.

## Introduction

Assessment of patient enablement may prove to be an important component of effective asthma care, but it is important to know about the applicability of existing instruments in different cultural settings.

Asthma is a chronic respiratory disease affecting 1–18% of the population.^1^ In Portugal, the latest estimate of the prevalence of ‘current asthma’ is 6.8%, similar in both men and women,^2^ whereas the estimate for ‘lifelong asthma’ is between 10.24 and 10.5%.^2,3^

International^1–6^ and national^7^ guidelines recommend the assessment of asthma control at every visit. The assessment should include symptom control and the risk of adverse outcomes, as well as a discussion of treatment issues, including inhaler technique, adherence, and the presence of adverse effects, and the management of comorbidities that contribute to the burden of symptoms and poorer quality of life.^1,6^

Despite the availability of asthma management guidelines and asthma therapies of proven efficacy, the majority of patients do not achieve good asthma control.^4,7–9^ In Portugal it is estimated that 43% of patients have uncontrolled asthma,^10^ similar to results from recent studies in other countries.^9^

The reasons for poor asthma control include errors in diagnosis, smoking, comorbid rhinitis, incorrect choice of inhalers, poor inhaler technique, individual variation in response to treatment, patient beliefs and concerns, treatment adherence, increased exposure to environmental allergens, decreased access to good care, suboptimal self-management and low socioeconomic status.^4,9–12^

Effective asthma management and improved outcomes require a partnership between the patient and healthcare providers.^1,4,8^ Appropriate asthma self-management^13,14^ may be the most cost-effective way to reduce morbidity and mortality.^1,9,15,16^ This includes monitoring symptoms, controlling allergens, adhering to treatment and adjusting medicines when necessary.^15^

Enabling the person with asthma to assume a major role in the management of their disease is cost effective and should be an integral component of any plan for long-term management of asthma, in accordance with international and national recommendations.^1,4^

The Patient Enablement Instrument (PEI) is a self-administered questionnaire that was developed in the United Kingdom.^17^ The PEI is related to, but different from, measures of satisfaction.^17^ This is a widely accepted tool that has been validated in many cross-sectional studies. It has been translated and evaluated in several countries, including Portugal. It has high internal consistency and delivers consistent results.^18–23^

In 2006, the PEI was adapted by Haughney and collaborators for use in asthma management, creating the modified PEI (mPEI).^24^ The mPEI demonstrated similar internal consistency and external validity to the original.^24^

The mPEI has since been used in the United Kingdom and Portugal.^24–26^ In a Portuguese study, the instrument was translated and culturally adapted but no psychometric properties were assessed, hence the importance of the present study.^26^

The aim was to adapt the mPEI to Portuguese asthma patients and ensure it is a valid and reliable instrument. So, the objectives of this study were to test the applicability of the mPEI to Portuguese asthma patients after translation and cross-cultural adaptation, and to verify the structural validity, internal consistency and reproducibility.

## Results

### Participants

The study included a random sample without replacement of 150 patients, aged 18 years old and over, with a diagnosis of persistent asthma. Initially a random group of 302 patients were approached for participation in the study and screened for exclusion criteria. From this group 80 patients had intermittent asthma and were not currently under chronic treatment with inhaler devices, 36 patients were unwilling to participate, 20 patients missed the first appointment, 10 patients were improperly diagnosed, 4 patients had cognitive impairment and 2 patients were unable to independently complete the questionnaire. In addition, 8 patients could not be reached (due to emigration and/or cancelling of telephone accounts). Although 150 patients were eligible for the study, only 142 patients could be recruited in time to participate in the study, 86 (60.6%) of whom completed the mPEI in both T1 and T2.

The sample consisted of 65 men and 77 women. The age of the participants ranged from 19 to 88. The demographic characteristics of the patients for both T1 and T2 are presented in Table 1.

The mean and s.d. of the mPEI (each item and total scale) at T1 and T2 are presented in Table 2. The total score of the mPEI at T1 had a minimum of 0 (1.40% of responses) and a maximum of 12 (19% of responses). There were no floor or ceiling effects. At T2, the total score of the mPEI had a minimum of 0 and a maximum of 12. Ceiling effect was observed in 24.40% of patients. There was no floor effect. The score for each item was significantly correlated with total mPEI score.

The suitability of data for factor analysis was assessed. Inspection of the correlation matrix revealed the presence of coefficients of 0.3 and above.

At T1, the KMO value was 0.85 and Bartlett’s Test of Sphericity reached statistical significance (*χ*^2^ (15)=402, *P*=0.000), which meant that the levels of correlation between the items of the instrument were high enough to perform factor analysis on the sample. PCA revealed the presence of 1 component with an initial eigenvalue of 3.68, explaining 61.3% of the variance, which consisted of all six items of the scale. Factor loadings are presented in Table 3. An inspection of the scree plot revealed a clear brake after the first component.

At T2, the KMO value was 0.82 and Bartlett’s Test of Sphericity reached statistical significance (*χ*^2^ (15)=295, *P*=0.000), so the levels of correlation between the items of the instrument were enough to perform factor analysis on the sample. PCA revealed the presence of 1 component with eigenvalue exceeding 1, explaining 63.1% of the variance, which consisted of all six items of the scale. Factor loadings are presented in Table 3. An inspection of the scree plot revealed a clear brake after the first component.

At T1, Cronbach’s alpha was 0.87, and 0.88 at T2. The values of item-total correlation were between 0.58 and 0.76, indicating that the mPEI has good internal consistency (Table 4). The mPEI revealed high test–retest reliability (ICC=0.85; confidence interval 95%; 0.78 to 0.90).

## Discussion

The purpose of this study was to perform a cross-cultural adaptation of the mPEI questionnaire to Portuguese and to examine the psychometric properties of the translated scale in a sample of Portuguese asthma patients. Careful linguistic translation is a necessary but not sufficient condition for the application of a patient-outcome instrument in different population, in a different culture, speaking a different language. The evaluation of psychometric properties is fundamental to support its cross-cultural application.

### Main findings

This study revealed good psychometric properties of the Portuguese mPEI. The results of the item-scale correlation and the principal component analyses performed using an exploratory approach support the internal construct validity of the mPEI as a measure of only one component—enablement. High correlations between items and the total score and principal factor demonstrated the relevance and importance of items.

The internal consistency was good suggesting the items measure the same construct and indicates that the scale is sufficiently reliable for use as an outcome variable in clinical research. Moreover, the instrument revealed high test–retest reliability, an aspect not tested in the original study or since. This is also supported by the fact that mPEI scores were reproducible between T1 and T2.^14,23^

The mean mPEI score in this study was higher than that previously reported in Portugal.^26^ There was a tendency for ceiling effects in T1 and in T2. Over 20% of patients scored the highest score, which could compromise the sensitivity of the instrument. However, as only 60.6% of patients participated in the data collection of T2, this could explain the observation. Patients who are more motivated and concerned with health issues may have a higher enablement score.

### Interpretation of findings in relation to previously published work

The Portuguese translation of the mPEI has a good correspondence to the original version, and the structure of the questions is simple and easily understood.^24^ The cultural adaptation did not present any difficulties, and the patients could comprehend the constructs involved.

### Strengths and limitations of this study

The responsiveness of the instrument, that is, the change in score over time in case of a meaningful change, is yet to be evaluated. Further studies are needed to help to establish it as an appropriate outcome measure in the long-term management of asthma.

### Implications for future research, policy and practice

The assessment of psychometric properties recommends the use for clinical practice of the Portuguese mPEI version.

### Conclusions

In conclusion, the Portuguese mPEI is semantically equivalent to the original instrument. It presented satisfactory measurement properties according to cross-cultural validity, reproducibility, internal consistency and factor analysis. Additional research will test its responsiveness and define it as a useful outcome measure to consider in the long-term management of asthma.

## Materials and methods

**Figure 1:**
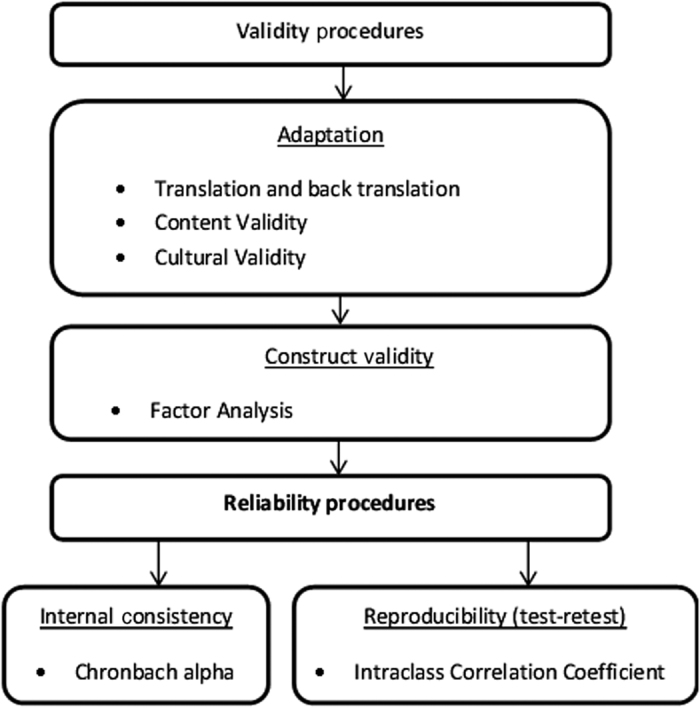
A flow chart depicting the process used for validation and reliability study of the Portuguese mPEI.

The methods used to adapt and validate the mPEI included the following: linguistic and cultural adaptation, construct validity and reliability tests. A flow chart depicting the processes used to examine the validity of the SQ is presented in Figure 1. Analyses were performed using Statistical Package for the Social Sciences (SPSS) software for Macintosh Operative System Version 22.0 (SPSS, IBM Company, Chicago, IL, USA).

### Translation and cultural validity

Permission from the authors of the mPEI was obtained in order to use and translate it into Portuguese. The translation was developed by forward–backward translations from the original English version. There were two independent translations, produced by two native Portuguese-speaking translators fluent in English. Both versions were compared by a panel of six general practitioners and, after consensus the instrument was prepared in a single document. This consensus version was then translated back to English, by two native English-speaking general practitioners fluent in Portuguese, without prior knowledge of the original version. This was then compared with the original, to ensure conceptual and semantic equivalence. Cognitive debriefing of the Portuguese version of the mPEI was performed with 10 asthma patients in order to evaluate the adequacy of the format and presentation and to assess the clarity and interpretation of each item and response option. Each item was understood and correctly interpreted by all patients. A panel consisting of six Family Physicians agreed that the Portuguese version demonstrated semantic and grammatical equivalence.

### Construct validity

The internal construct validity of the scale was first evaluated by Spearman’s correlation coefficient between the item and the scale score. An item should be substantially linearly related (*ρ*⩾0.4) to the underlying concept (total score) being measured.^27,28^ To verify construct validity, the factorial design of the Portuguese version of the mPEI was analysed in a stepwise procedure. At both T1 and T2, two measures were used to assess sampling adequacy—Kaiser–Meyer–Olkin measure (KMO) and Bartlett’s test of sphericity. KMO is a measure of sampling adequacy calculated for both the entire correlation matrix and each variable individually. KMO values above 0.60 indicate that the sample is adequate for factor analysis.^27,28^ Bartlett’s test indicates the strength of the association between variables. It tests the null hypothesis that there is no association among the variables, only of each variable with itself. Factor analysis is only indicated when the null hypothesis is rejected. Assuming adequacy of the sample, a principal component analysis (PCA) with varimax rotation was also conducted. The number of factors extracted was determined using the Kaiser method, i.e., according to initial eigenvalues (retention if >1.0) and the visualisation of the Cattell’s scree plot in order to assess the amount of variance accounted for by a factor.^27,28^ All factor loadings (i.e., correlations of an item with a factor) greater than 0.50 were considered relevant.^27,28^

### Reliability study

The temporal stability of both scales was assessed by test–retest by asking participants to complete both instruments 7 days after the first interview. The change in mean scores between the test and the retest was evaluated by the intra-class correlation coefficient (ICC) with values ranging from 0 (no stability) to 1 (perfect stability).^27,28^ Cronbach’s alpha was used to examine the internal consistency of the instrument. It was considered that values >0.70 represent acceptable consistency.^27,28^ Corrected item-total correlation of 0.30 or higher was considered acceptable for each item.^27,28^

### Participants

This study was conducted in two Family Health Units (FHU), in Matosinhos, Portugal during February and March 2015. The sample was randomly selected without replacement from the local database of 620 asthma patients. Asthma was classified as persistent according to the indication for chronic therapy with inhaled corticosteroids. The sample size was calculated to assure 20 observations per item.^23^ Patients with cognitive impairment, kyphoscoliosis, absence of one lung, lung cancer, pregnancy and those unable to provide informed consent, or unable to independently complete the questionnaire were excluded before randomisation. Randomisation was performed using the number generator tool from SPSS. First, 10 patients were randomly selected to assess translation and cultural validation. These patients were later excluded from the randomisation. Second, 150 patients were randomly selected for interview. No financial compensation was provided for participation. The study was explained to each patient by telephone. The patients who agreed to participate were invited to come to the FHU at a time of their choosing for an interview in order to verify their eligibility to enter the study and to review the consent form. During the interview, demographic data, including gender, age and educational level, were collected on a form, identified with a unique number to preserve confidentiality. Each patient was scheduled for a second interview 7 days later (T2), at the FHU, at a time of their choosing.

### Questionnaire: modified patient enablement instrument

The mPEI is a six-item questionnaire. Each item is scored in a Likert-type scale from 0 (‘same or less’ or ‘not applicable’) to 2 (‘much better’). There is a minimum score of 0 and a maximum score of 12. A higher score reflects higher patient enablement. The mPEI has good internal consistency with a reported Cronbach’s alpha coefficient of 0.92.^14^ The translated version to Portuguese may be found in Supplementary Appendix 1.

### Ethical approval

This study was approved by the Ethics Sub-committee for the Health and Life Sciences of University of Minho and by the Ethics Committee of the Local Health Authority in Matosinhos (053/CE/JAS).

## Figures and Tables

**Table 1:** Characteristics of participants at T1 and T2 (7 days later)

*Characteristics of patients*	*T1*^a^ (n*=142)*	*T2*^b^ (n*=86)*	P-*value*
Male/female, *n* (%)	65 (45.8%)/77 (54.7%)	33 (38.4%)/53 (61.6%)	0.30
			
*Age*			
Mean±s.d.	49.3±17.0 years	50.8±17.1 years	0.52
Skewness (s.e.)	−0.04 (0.20)	−0.15 (0.26)	
Kurtosis (s.e.)	−0.99 (0.40)	−0.95 (0.51)	
			
*Education level*			
⩽4 years, *n* (%)	53 (37.3%)	37 (43%)	0.39
6 years, *n* (%)	9 (6.30%)	5 (5.80%)	0.88
9 years, *n* (%)	17 (12%)	7 (8.10%)	0.35
11 years, *n* (%)	6 (4.20%)	3 (3.50%)	0.79
12 years, *n* (%)	26 (18.3%)	14 (16.3%)	0.70
>12 years, *n* (%)	31 (21.8%)	20 (23.3%)	0.79

a First interview.

b Second interview (7 days after first interview).

**Table 2:** Item and scale descriptives and item-scale correlations of the Portuguese mPEI at T1 and T2 (7 days later)

*mPEI item*	*T1*^a^ (N*=142)*			*T2*^b^ (N*=86)*		
	*Mean*	*s.d.*	*Correlation with total mPEI score*	*Mean*	*s.d.*	*Correlation with total mPEI score*
Item 1	1.23	0.71	*ρ* (140)=0.77*	1.34	0.68	*ρ* (84)=0.84*
Item 2	1.39	0.67	*ρ* (140)=0.75*	1.50	0.61	*ρ* (84)=0.79*
Item 3	1.43	0.64	*ρ* (140)=0.80*	1.50	0.59	*ρ* (84)=0.80*
Item 4	1.26	0.68	*ρ* (140)=0.83*	1.37	0.60	*ρ* (84)=0.79*
Item 5	1.20	0.66	*ρ* (140)=0.84*	1.31	0.64	*ρ* (84)=0,77*
Item 6	1.33	0.65	*ρ* (140)=0.71*	1.41	0.64	*ρ* (84)=0.77*
Total	7.85	3.13		8.43	2.98	

a First interview.

b Second interview (7 days after first interview). *P<0.05.

**Table 3:** Principal component analysis of the Portuguese mPEI for T1 and T2 (7 days later)

*T1*^a^			*T2*^b^	
*mPEI*	*Loading factors*	*Communalities*	*Loading factors*	*Communalities*
Item 1	0.85	0.57	0.84	0.71
Item 2	0.83	0.55	0.81	0.66
Item 3	0.81	0.66	0.83	0.68
Item 4	0.76	0.69	0.78	0.62
Item 5	0.75	0.71	0.76	0.58
Item 6	0.71	0.50	0.74	0.54

a First interview.

b Second interview (7 days after first interview).

**Table 4:** Internal consistency of the Portuguese mPEI for T1 and T2 (7 days later)

*mPEI*	*T1*^a^		*T2*^b^		*ICC CI (95%)*		
	*Corrected Item-Total Correlation*	*Cronbach's Alpha if Item Deleted*	*Corrected Item-Total Correlation*	*Cronbach's Alpha if Item Deleted*	*ICC*	*Lower*	*Upper*
Item 1	0.64	0.86	0.75	0.85	0.67	0.50	0.79
Item 2	0.63	0.86	0.71	0.86	0.63	0.44	0.76
Item 3	0.71	0.84	0.73	0.86	0.78	0.66	0.86
Item 4	0.73	0.84	0.67	0.86	0.78	0.67	0.86
Item 5	0.76	0.84	0.66	0.87	0.77	0.65	0.85
Item 6	0.58	0.87	0.63	0.87	0.69	0.53	0.80

		Cronbach’s alpha		Cronbach’s alpha	ICC	Lower	Upper
Score		0.87		0.88	0.85	0.76	0.90

Abbreviation: CI, confidence interval.

a First interview.

b Second interview (7 days after first interview).
