# Supplementary figures and images for: The modified patient enablement instrument: a Portuguese cross-cultural adaptation, validity and reliability study
Source: NPJ Prim Care Respir Med. 2017 Jan 12;27:16087–. doi: 10.1038/npjpcrm.2016.87 (PMC5228497; doi:10.1038/npjpcrm.2016.87)

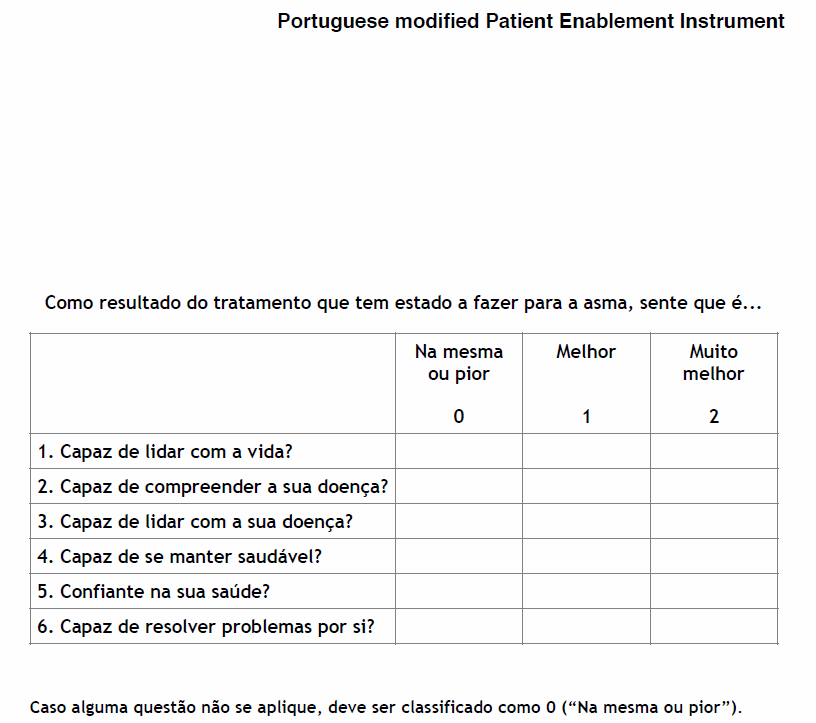

Supplement: Supplementary Appendix 1 [file npjpcrm201687-s1.tiff]
